# Methylation profile of the *ADRB3* gene and its association with lipid profile and nutritional status in adults

**DOI:** 10.1186/s40659-019-0226-7

**Published:** 2019-04-06

**Authors:** Raquel Patrícia Ataíde Lima, Marina Ramalho Ribeiro, Keylha Querino de Farias Lima, Elisama Araújo de Sena, Diego de Oliveira Costa, Rafaella Cristhine Pordeus Luna, Rayner Anderson Ferreira do Nascimento, Maria da Conceição Rodrigues Gonçalves, Rodrigo Pinheiro de Toledo Vianna, Ronei Marcos de Moraes, Naila Francis Paulo de Oliveira, Aléssio Tony Cavalcanti de Almeida, Maria José de Carvalho Costa

**Affiliations:** 1 Graduate Program in Nutrition Sciences, Health Sciences Center (Centro de Ciências da Saúde, CCS), Federal University of Paraíba (UFPB), João Pessoa, Brazil; 2 Graduate Program in Molecular and Human Biology, Natural Exact Sciences Center (Centro de Ciências Exatas da Natureza, CCEN), UFPB, João Pessoa, Brazil; 3 Graduate Program in Nutrition, CCS, Federal University of Paraíba - UFPB), João Pessoa, Brazil; 4 Graduate Program in Health Decision Models, CCEN, UFPB, João Pessoa, Brazil; 5 Department of Economics, Applied Social Sciences Center (Centro de Ciências Sociais Aplicadas CCSA), UFPB, João Pessoa, Brazil

**Keywords:** Epigenetics, DNA methylation, Obesity, Lipid, *ADRB3*

## Abstract

**Background:**

Defects in DNA methylation have been shown to be associated with metabolic diseases such as obesity, dyslipidemia, and hypercholesterolemia. To analyze the methylation profile of the *ADRB3* gene and correlate it with lipid profile, lipid intake, and oxidative stress based on malondialdehyde (MDA) and total antioxidant capacity (TAC), homocysteine and folic acid levels, nutritional status, lifestyle, and socioeconomic variables in an adult population. A cross-sectional epidemiological study representative of the East and West regions of the municipality of João Pessoa, Paraíba state, Brazil, enrolled 265 adults of both genders. Demographic, lifestyle, and socioeconomic questionnaires and a 24-h recall questionnaire were applied by trained interviewers’ home. Nutritional and biochemical evaluation (DNA methylation, lipid profile, MDA, TAC, homocysteine and folic acid levels) was performed.

**Results:**

DNA hypermethylation of the *ADRB3* gene, analyzed in leukocytes, was present in 50% of subjects and was associated with a higher risk of being overweight (OR 3.28; p = 0.008) or obese (OR 3.06; p = 0.017), a higher waist–hip ratio in males (OR 1.17; p = 0.000), greater intake of trans fats (OR 1.94; p = 0.032), higher LDL (OR 2.64; p = 0.003) and triglycerides (OR 1.81; p = 0.031), and higher folic acid levels (OR 1.85; p = 0.022).

**Conclusions:**

These results suggest that epigenetic changes in the *ADRB3* gene locus may explain the development of obesity and non-communicable diseases associated with trans-fat intake, altered lipid profile, and elevated folic acid. Because of its persistence, DNA methylation may have an impact in adults, in association with the development of non-communicable diseases. This study is the first population-based study of the *ADRB3* gene, and the data further support evaluation of *ADRB3* DNA methylation as an effective biomarker.

## Introduction

DNA methylation is the most well-known epigenetic modification, and it is performed by the enzyme family of DNA methyltransferases (DNMTs), which catalyze the transfer of a methyl group from *S*-adenosylmethionine (SAM) to the 5′-carbon of a cytosine that precedes guanine (CpG dinucleotide) [1]. Methylation frequently occurs in gene promoter regions, which are often rich in CpG dinucleotides and are thus called CpG islands. DNA hypermethylation inhibits gene transcription by preventing the binding of transcription factors or by binding methyl-CpG-binding proteins (MBPs) [2].

Previous studies suggest that DNA methylation plays an important role in metabolic diseases such as obesity, dyslipidemia, and hypercholesterolemia [3, 4].

Epigenetics involves hereditary changes in gene expression that do not involve changes in the DNA sequence; these changes are reversible and may be repetitively transferred to subsequent generations [5, 6]. The three main characteristics of epigenetics are: (1) no changes in DNA sequence, (2) inheritability, and (3) plasticity and reversibility [7].

Unlike the genome, the epigenome can be modified, and thus some epigenetic risk markers have the potential to be removed. These modifications can be caused by medications, diet, or exposure to environmental factors [8]. These factors, when associated with oxidative stress and inflammation, tend to influence the DNA methylation profile or histone modifications [9]. This specific knowledge may lead to the development of potential therapeutic targets for the prevention and treatment of non-communicable diseases.

On the other hand, it is known that genes involved in metabolism can contribute to differences among individuals in regard to nutrient requirements and susceptibility to disease [10]. One of these genes is *ADRB3*, beta-3 adrenergic receptor, which has been associated with dyslipidemia [11]. *ADRB3* is located on chromosome 8 and contains a CpG island in its promoter region. *ADRB3* is expressed in the white and brown adipose tissues [12] and in peripheral lymphocytes [13], also is involved in lipolysis and thermogenesis regulation. Only two studies have evaluated the methylation profile of *ADRB3* in an obese population. One of the studies showed that *ADRB3* expression is lower in this population [2, 14]. The decreased expression of this gene may lead to poor performance in lipolysis regulation in human brown and white adipose tissues that provide free fatty acids for thermogenesis, i.e., there is indirect evidence from the studies conducted that *ADRB3* participates in the regulation of body weight in humans [14].

Studies evaluating the importance of the association of the DNA methylation profile with lifestyle (dietary habits, alcohol consumption, smoking, and physical activity), biochemical values, and nutritional status in adults should promote new research to support intervention strategies in the fight against non-communicable diseases [15, 16].

In this context, we describe the first population-based study of the *ADRB3* gene to analyze the *ADRB3* methylation profile and its association with nutritional status, lipid intake, lipid profile, and biochemical, lifestyle, and socioeconomic markers in adults of both genders.

## Methods

### Study design

This cross-sectional epidemiological study is linked to the project titled: “II Cycle of Diagnosis and Intervention of Food and Nutritional Status and the Most Prevalent Non-Communicable Diseases in the Population of the Municipality of João Pessoa/PB” (II Ciclo de Diagnóstico e Intervenção da Situação Alimentar, Nutricional e das Doenças não Transmissíveis mais Prevalentes da População do Município de João Pessoa/PB, II DISANDNT/PB). Data were collected between May 2015 and May 2016.

### Ethical issues

This study was approved by the Research Ethics Committee of the Health Sciences Center (Centro de Ciências da Saúde, CCS) of the Federal University of Paraíba (Universidade Federal da Paraíba, UFPB) under protocol number 0559/2013 in accordance with the ethical standards for research involving human beings included in Resolution 466 of the December 12, 2012 meeting of the National Health Council/National Research Ethics Committee. Participants provided consent by signing an informed consent form.

### Sampling

To conduct this population-based study that was representative of the East and West regions of the municipality of João Pessoa, a sample that was representative of the adult group was calculated using information provided by the city hall, such as the city map, number of blocks per neighborhood, and data from the Brazilian Institute of Geography and Statistics (Instituto Brasileiro de Geografia e Estatística, IBGE) [17].

For the sample calculation, a single stratified sampling procedure was used. Given the heterogeneity of the variable “income” and the relationship between income, disease prevalence, and nutrition [18], a stratified sampling approach was used [19] for the blocks at a first level. At this level, the neighborhoods of the East and West regions of the municipality were classified into four income strata according to information obtained from the IBGE [17].

**Fig. 1:**
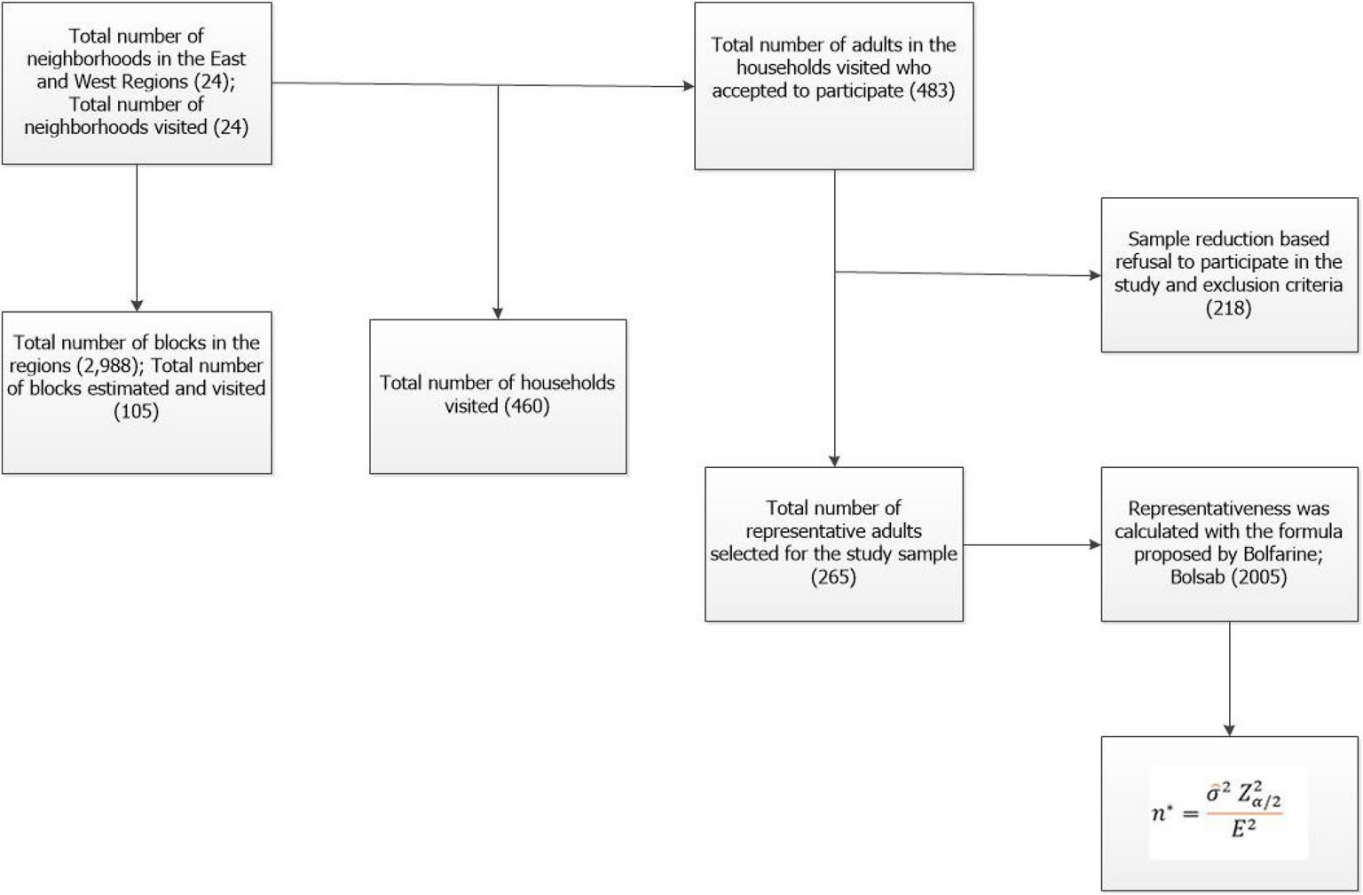
Diagram for sample definition—East and West Regions of the Municipality of João Pessoa

All the neighborhoods (24) of the East and West regions of the municipality of João Pessoa were visited, totaling 2988 blocks, with an estimate of visits for 105 blocks (Fig. 1). After determining the number of blocks to be sampled in each neighborhood, a random drawing was performed. For this purpose, the city map with the numbered blocks was used, and random numbers with uniform distribution were generated using a pseudorandom number generator and the Core R Development Team (2006) software for selection. The diagram for the definition of the sample representative of the East and West regions of the municipality of João Pessoa/PB is shown in Fig. 1.

This study had the following as inclusion criteria: individuals aged 20–59 years; different socioeconomic conditions, and medication users and non-users. Exclusion criteria included the following: individuals with neuropsychiatric disorders; multivitamin, mineral, anorexigen, and anabolic supplement users; and pregnant and lactating women.

### Data collection

The household visits and application of the questionnaires were conducted by teams of researchers who were trained before initiating data collection after the pilot study was conducted. The trained teams, upon recognition of the blocks selected, were instructed to select all the households in the block. At each household selected, all the adults aged 20–59 years were invited to participate in the study through the application of questionnaires for demographic, socioeconomic, and epidemiological characterization and nutritional and food intake assessment, and biochemical evaluation and *ADRB3* gene methylation profiling were performed with consent signed.

### Nutritional assessment

Weight and height measurements were taken in triplicate, and the average of the three values was used. The World Health Organization (WHO) recommends the following cutoffs of body mass index (BMI) to classify the nutritional status of adults between 20 and 59 years of age: < 18.5 kg/m^2^ (underweight), 18.5–24.9 kg/m^2^ (normal weight), 25.0–29.9 kg/m^2^ (overweight), 30.0–39.9 kg/m^2^ (obesity), and ≥ 40 kg/m^2^ (morbidly obese) [20].

The indicators used to determine the abdominal obesity status were waist circumference and waist–hip ratio (WHR). The American Heart Association recommends cutoffs of waist circumference to determine the abdominal obesity status of ≥ 102 cm in men and ≥ 88 cm in women [21]. According to the WHO, abdominal obesity is defined as a WHR > 0.90 in men and > 0.85 in women [20].

### Dietary assessment

Food intake data were obtained through interviews conducted at the participant’s house on two business days and one weekend day, using 24-h recall. More specifically, all the participants in the study were asked to describe the type and amount of food and drinks consumed the day before.

In order to effectively quantify the consumed portion sizes and to minimize potential memory biases of the individuals studied [22–24]. Common household measures (cups, spoons, etc.) were used, and a book with images of food depicted in household measures in four sizes (small, medium, large, and extra-large) were used to ascertain the actual weight of the food products consumed.

The food data were converted into the respective macro and micronutrient values and analyzed using DietWin software version 2013. For the assessment of nutrient intake, only complete 24-hour recalls with energy intake between 500 and 5000 kcal/day were considered [25, 26]. Nutrient intake data were adjusted for inter and intra-individual variations using the Multiple Source Method (MSM) available online [27] (https://msm.dife.de/tps/msm/).

### Individual energy intake calculation

Calorie intake was calculated individually and for each gender, according to the formula proposed by OTTEN et al. [28]. For men, EER = 662 − (9.53 × age (years) + PA × [(15.91 × weight (kg)) + (539.6 × height (m))], and for women, EER = 354 − (6.91 × age (years) + PA × [(9.36 × weight (kg)) + (726 × height (m))], where EER is the estimated energy requirements, and PA is the physical activity factor.

### Biochemical tests

Blood was collected from fasting subjects on the third day of household visits. The blood was collected by an experienced nurse.

Vacutainers^**®**^ were used (two 4-mL tubes) for the blood collection. One was for serum analysis (tube with clot activator), and the other was for plasma analysis (with ethylenediaminetetraacetic acid [EDTA] anticoagulant). The tubes were maintained inside a thermal box during blood collection, where they remained for a maximum of 2 h.

Lipid profiling was performed using kits commercial (Labtest^®^ Brazil) to measure total cholesterol through the Trinder enzymatic method, high-density lipoprotein cholesterol (HDL-C) through the precipitation method, and triglycerides through the Trinder enzymatic method. Values for plasma low-density lipoprotein cholesterol (LDL-C) were estimated by the equation proposed [29], where (LDL-C = Total Cholesterol − HDL-C − Triglycerides/5). Malondialdehyde (MDA) was quantified through the reaction of thiobarbituric acid (TBA) with hydroperoxide decomposition products, as described by Ohkawa, Ohishi, and Yagi [30].

The total antioxidant capacity (TAC) was measured with DPPH (1.25 mg diluted in 100 mL of ethanol and protected from light) [All tests were performed at the Research Laboratory of Physical Training Applied to Performance and Health (Laboratório de Estudos do Treinamento Físico Aplicado ao Desempenho e a Saúde, LETFADS) at the Department of Physical Education of the Federal University of Paraíba].

Homocysteine and folic acid concentrations were measured at the Roseane Dore Clinical Laboratory. Homocysteine was quantified with the method described by Pfeiffer et al., using high-performance liquid chromatography (HPLC) with fluorescence detection and isocratic elution [31] with an analytical sensitivity of 1.74 µmol/L. Folic acid was analyzed with the chemiluminescence method, considering the reference value > 3.10 ng/mL and an analytical sensitivity of 0.5 ng/mL.

### DNA methylation profile

#### Sample extraction and isolation of genomic DNA

Genomic DNA was extracted from the leukocytes using TRIZOL LS (Life Technologies, California, USA) according to the manufacturer’s recommendations. Genomic DNA was quantified with a Nanodrop^®^ spectrophotometer (Thermo Scientific, California, USA) and stored at − 20 °C until further analysis.

#### DNA methylation analysis of the ADRB3 gene promoter

**Fig. 2:**
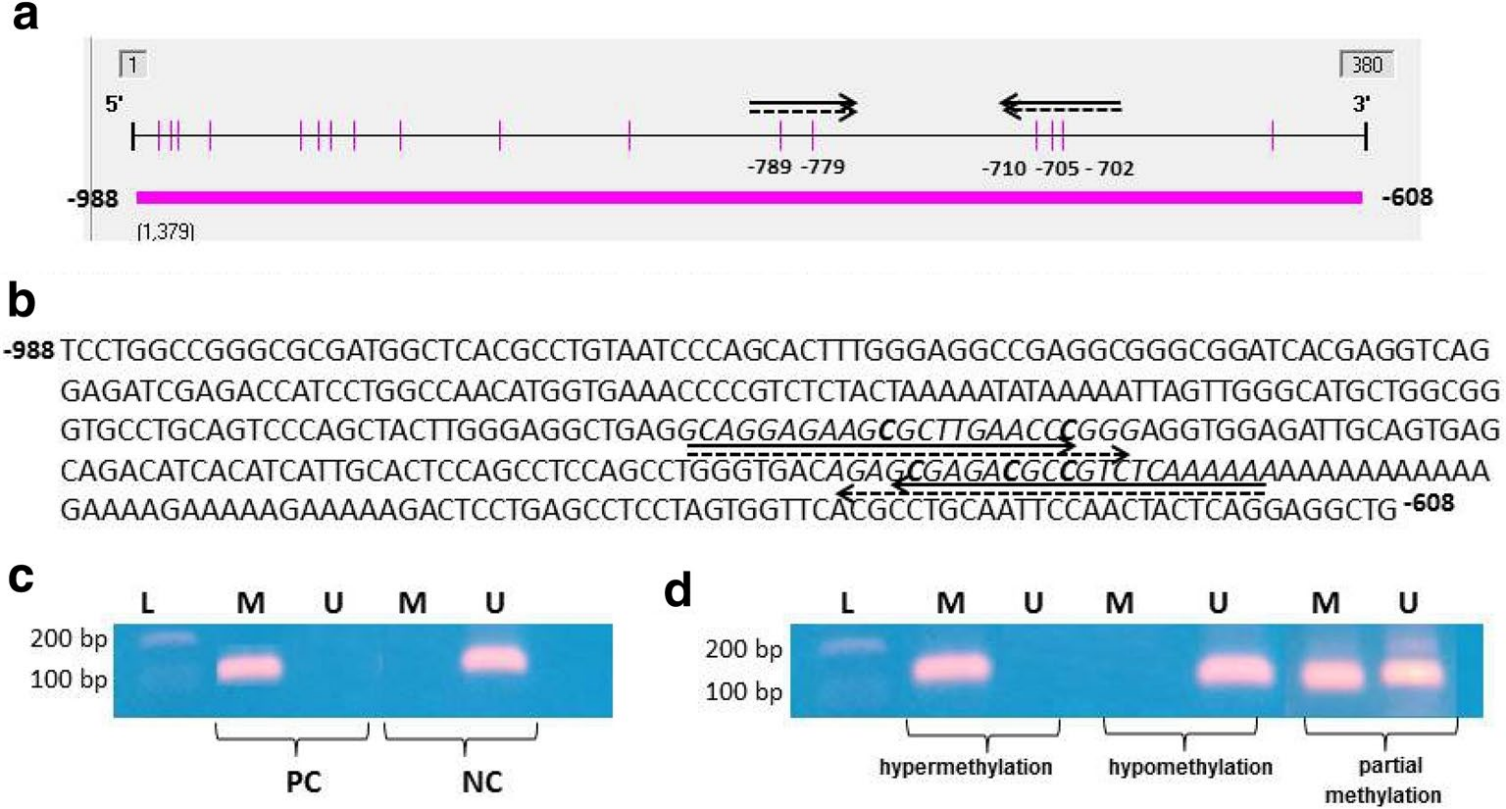
**a** Schematic and **b** partial genomic sequence of the CpG island in the promoter region of *ADRB3* ranging from − 988 to − 608 bp (GenBank accession number DQ104441.1). The CpG site studied is presented. Primer regions are indicated by arrows (solid for methylated sequence and dashed for non-methylated sequence). Horizontal bar—CpG island; vertical bars—CpG sites. **c** Bands of positive (methylated) and negative (unmethylated) control DNA, showing specific amplification for methylated and unmethylated conditions after polymerase chain reaction (109 bp). **d** Bands of representative samples of the three conditions found in the study population: hypermethylation, hypomethylation, and partial methylation. Partially methylated DNA refers to the amplified samples for methylated and unmethylated conditions indicating that methylation does not occur in all cells or alleles. L- DNA ladder; *M* methylated, *U* unmethylated, *PC* positive control, *NC* negative control

Methylation analysis was performed using methylation-specific PCR (MSP). Genomic DNA (800 ng) was modified with sodium bisulfite using the Cells-to-CpG™ Bisulfite Conversion Kit (Life Technologies) according to the manufacturer’s specifications. The CpG sites studied are located in a CpG island in the promoter region spanning nucleotides − 807 to − 747, as confirmed by the Methyl Primer Express v.1.0 software (Applied Biosystems) from data deposited in GenBank. The differences in DNA sequences after treatment were detected by PCR using primers specific for methylated (F: 5′-GTAGGAGAAGCGTTTGAATTC-3′, R: 5′-TTTTTTAAAACGACGTCTCG-3′) and unmethylated DNA (F: 5′-GAGGTAGGAGAAGTGTTTGAATTT-3′, R:5′-TTTTTTTAAAACAACATCTCACT-3′) sequences, generating a 109-bp fragment (GenBank accession number DQ104441.1) (see Fig. 2). Each MSP reaction included 100 ng of bisulfite-modified DNA, 1 µL (10 µM) of each primer and Go Taq Hot Start Green Master Mix 1× (Promega Corporations, Madison, WI, USA) in a 25-µL of final reaction volume. The conditions for fragment amplification were as follows: 95 °C × 5 min; 40 cycles of 95 °C × 1 min, 60 °C − methylation conditions/55 °C − non-methylation conditions − × 1 min, and 72 °C × 7 min, and a final elongation at 72 °C × 5 min. Following amplification, 10 µL of the PCR samples were loaded onto a 10% polyacrylamide gel for electrophoresis and stained with Gel Red (Biotium). Methylated and unmethylated DNA (Cells-to CpG™ Methylated and Unmethylated gDNA Control Kit, Life Technologies) was modified as previously described and amplified by PCR for use as positive controls (see Fig. 2).

## Statistical analysis

An initial descriptive analysis of the characteristics of the sample represented by simple frequencies was performed using measures of central tendency and dispersion (mean, standard deviation, and median). The data were evaluated for normality using the Lilliefors test, a modification of the Kolmogorov–Smirnov test [32]. Statistical analysis was conducted with STATA 13 software [33]. To calculate nonlinear regression, odds ratio was used, and the median of the continuous variables was used to identify the coefficient’s signal and the existence of a significant association between the variables. All variables with p value < 0.05 were included in the model and considered significant.

## Results

Data on anthropometric, food intake, and biochemical characteristics of the study are presented with the methylation status of *ADRB3* in Table 1. The total study population was predominantly women, and more than half of the adults were overweight and obese based on the BMI. Approximately 50% of the population studied presented hypermethylation of *ADRB3* (Table 1).

**Table 1:** General characteristics of the adult individuals

	Mean	Standard deviation	Median	N	%
Demographic, socioeconomic and lifestyle characteristics					
Gender					
Male	–	–	–	79	30
Female	–	–	–	186	70
Menopausal women (≥ 50 years)^a^				61	32.8
Age					
20–59 years	40.3	14.3	39.5	265	100
Education level^b^					
Complete elementary school	–	–	–	56	21.13
≥ Incomplete middle school	–	–	–	209	78.9
Household income ($)^c^	1458.43	1595.5	820.51	265	100
Alcohol consumption					
Yes	–	–	–	42	15.8
No	–	–	–	223	84.2
Smoker					
Yes	–	–	–	21	8.0
No	–	–	–	244	92.0
Physical activity (150 min/week)					
Yes				108	40.75
No				157	59.24
Days per week	4.5	1.4	4.5	108	48.0
Duration of activity, min/week	403	220	360	108	40.75
Anthropometric characteristics					
Weight (kg)	72.11	16.74	71.6		
Height (m)	1.63	0.09	1.62		
BMI (kg/m^2^)	27.08	5.88	26.71		
Waist (cm)	0.86	0.15	0.86		
Hip (cm)	1.02	1.02	12.3		
Nutritional classification (kg/m^2^)^d^					
Underweight/normal weight	21.8	2.37	22.36	124	46.8
Overweight	27.63	1.33	27.62	82	31
Obese	34.63	4.95	32.81	59	22.2
Food intake					
Calories (kcal)	1678	390.6	1625		
Total fat (g)	54.92	18.44	50.58		
Cholesterol (mg)	216.94	125.82	187.16		
Saturated Fat (g)	16.99	6.51	16.58		
Monounsaturated Fat (g)	14.39	5.33	13.39		
Polyunsaturated Fat (g)	13.64	7.55	12.1		
Oleic acid (g)	10.23	4.47	9.32		
Omega-6 (g)	6.30	2.70	6.07		
Omega-3 (g)	0.70	0.44	0.59		
Trans Fat (g)	0.65	0.4	0.56		
Folate (mcg)	130.0	22	144.0		
Biochemical Tests					
Homocysteine (μmol/L)					
Men (RV: 6.42 to 18.08)	11	9.8	6.6		
Women (RV: 5.32 to 15.22)	10	8.5	5.95		
Folic acid (RV: > 3.10 ng/mL)	13.5	5.1	12.5		
Lipid profile (mg/dL)					
Cholesterol (desirable VR: < 200)	184.2	42.5	178		
LDL (desirable RV: 100–129)	103.5	51.4	95		
HDL (desirable RV: > 60)	41.2	10.6	40		
Triglycerides (desirable RV: < 150)	140	87.7	111		
Oxidative stress					
TAC (%) (RV: 0–100%)	41	14	42		
MDA (RV: 2.3 to 4.0 µmol/L)	2.7	0.7	2.7		
DNA methylation					
ADRB3					
Unmethylated	–	–	–	56	21.13
Methylated	–	–	–	133	50.18
Partially methylated	–	–	–	76	28.7
Total				265	100

*RV* reference value, *BMI* body mass index, *TAC* total antioxidant capacity, *MDA* malondialdehyde

^a^According to the Ministry of Health (2008)

^b^Completed elementary school corresponds to 9 years of school, and ≥ complete elementary school corresponds to more than 10 years of school

^c^The income was based on the value of 3.90 BRL per USD

^d^According to the WHO [48]

In Table 2, the nutritional status and the median values for regular energy, total fat, saturated fat, monounsaturated fat, polyunsaturated fat, cholesterol, and trans-fat intake were analyzed for the hypermethylated individuals. The individuals who were overweight had a 228% great chance of *ADRB3* hypermethylation (OR 3.28), and obese individuals had a 206% great chance of hypermethylation (OR 3.06); men with a WHR > 0.90 cm had a 17% great chance of hypermethylation (OR 1.17), and individuals with a trans-fat intake above the median had a 94% (OR 1.94) higher chance of hypermethylation. For folate intake, the OR was lower than 1 and was not considered a risk factor.

**Table 2:** Odds ratio (95% CI) for the association of nutritional status, lifestyle, and food intake according to *ADRB3* hypermethylation of adult individuals

All the individuals (men and women)	*Hypermethylated ADRB3*		95% CI	
All the individuals	OR	p value	LL	UL
Normal weight (kg/m^2^)^a^	1.45	0.397	0.6135	3.4311
Overweight (kg/m^2^)^a^	3.28	0.008*	1.3668	7.8767
Obese (kg/m^2^)^a^	3.06	0.017*	1.2246	7.6582
Waist–hip ratio (men) (cm/cm)	1.17	0.000*	0.0639	2.4535
Waist–hip ratio (women) (cm/cm)	1.18	0.568	0.5942	1.3/168
Alcohol consumption	0.16	0.641	0.5332	0.8660
Physical activity	0.44	0.548	0.3548	0.6683
Calories (kcal)				
> Median	1.32	0.364	0.7190	2.4564
Total fat (g)				
> Median	0.92	0.854	0.4088	2.0979
Saturated fat (g)				
> Median	1.77	0.170	0.7809	4.0479
Polyunsaturated fat (g)				
> Median	0.65	0.142	0.3672	1.1543
Monounsaturated fat (g)				
> Median	0.79	0.524	0.3854	1.6246
Cholesterol (mg)				
> Median	1.60	0.113	0.8934	2.8944
Trans fat (g)				
> Median	1.94	0.032*	0.2658	2.0363
Folate (mcg)				
> Median	0.56	0.132	0.2344	1.2345

Also conducted with omega-3 and omega-6 intake but without significant difference

*CI* confidence interval, *LL* lower limit, *UL* upper limit, *OR* odds ratio

* p < 0.05

^a^(WHO [48])

In Table 3, the median values of blood lipids, oxidative stress, homocysteine, and folic acid were also used, and an association with cholesterol values greater than the median was observed, which indicates that individuals with values above the median present a 1 [27] % greats chance of having *ADRB3* hypermethylation (OR 2.27).

**Table 3:** Odds ratio (95% CI) for the association of biochemical test results with *ADRB3* hypermethylation of the adult individuals

All the individuals (men and women)	Hypermethylated *ADRB3*		95% CI	
All the individuals	OR	p value	LL	UL
Cholesterol (mg/dL)				
> Median	2.27	0.010*	0.2357	2.8182
LDL (mg/dL)				
> median	2.64	0.003*	1.3959	4.9970
HDL (mg/dL)				
> Median	0.62	0.101	0.3572	1.0960
Triglycerides (mg/dL)				
> Median	1.81	0.031*	1.056	3.1099
MDA (µmol/L)				
> Median	1.02	0.938	0.6089	1.7109
TAC (%)				
> Median	0.73	0.233	0.4383	1.2224
Homocysteine (µmol/L)				
> Median	0.82	0.484	0.4924	1.3985
Folic acid (ng/mL)				
> Median	0.53	0.022*	0.0926	2.1429

* p < 0.05

LDL above the median had a 164% higher chance of *ADRB3* hypermethylation, whereas individuals with triglyceride values above the median had an 81% (OR 1.81) higher chance of hypermethylation, further, individuals with folic acid values below the median had a 85% higher chance of hypermethylation.

In Table 4, the prevalence of inadequate energy; total, saturated, monounsaturated, polyunsaturated, and trans-fat; cholesterol, and folate intake is shown. The majority of the adults had a calorie intake below the recommended intake (80.6%), and total, monounsaturated, and polyunsaturated fat intakes were within the recommended range. With regard to saturated fat intake, the majority of subjects reported intake below the recommended value, and the same situation was observed for folate intake.

**Table 4:** Prevalence of inadequate intake of regular energy; total, saturated, monounsaturated, polyunsaturated, and trans fat; cholesterol; and folate

Nutrient	Average regular intake	Recommended intake	> Recommended intake (%)	< Recommended intake (%)	Intake within the reference range
Calories					
Men	1834.78 kcal	2855^ad^	03 (3.8)	70 (80.6)	06 (7.6)
Women	1611.46 kcal	2208^ad^	14 (7.5)	145 (54.7)	27 (14.5)
Total fat	54.92 g	25–35%^b^	49 (18.5)	22 (8.3)	194 (73.2)
Saturated fat	9%	< 10%^b^	97 (36.6)	168 (63.4)	–
Monounsaturated fat	14.39%	< 20%^b^	–	–	265 (100)
Polyunsaturated fat	13.64%	< 10%^b^	35 (13.2)	–	230 (86.8)
Folate^b^ (µg)	130	320^c^	34 (12.8)	200 (75.5)	31 (11.7)

^a^The recommendations were evaluated individually, according to the formula proposed by OTTEN et al. [28]

^b^American Heart Association [49]

^c^According with the Dietary Guidelines for Americans [50]

^d^Average recommended intake

## Discussion

The results of this study demonstrate that *ADRB3* gene methylation was present in half of the population studied; the majority of the population were women, overweight or obese, had an average income of 1458.43 USD, and completed middle school.

This methylation profile was associated with being overweight and obese for both genders and with a higher waist–hip ratio for men. In regard to regular nutrient intake, hypermethylation was only associated with a trans-fat intake above the median, and when correlated with the lipid profile, there was a significant association with cholesterol, LDL, and triglyceride levels above the median, in addition to an association with folic acid values below the median.

Hypermethylation of the *ADRB3* gene promoter in blood cells and visceral fat tissue has previously been associated with the development of obesity and metabolic complications in men [2]. By contrast, a different study revealed that there is no association between obesity and *ADRB3* hypermethylation in the fat tissue of obese men [14], but it is not clear in that study which CpG site in the promoter was studied, and decreased *ADRB3* expression was observed in this population. It is known that methylation profiles can vary in different tissues [34] and CpG sites [35], and it can also vary for populations that differ in intrinsic factors such as age or gender [36, 37] and extrinsic factors such as diet and smoking [15, 35], which may explain the difference between the studies.

In the study conducted by Oliveira et al. [38], it was observed that higher levels of methylation were not associated with lipid profile and oxidative stress, when the individuals were separated according to eutrophic, overweight and obesity classification, the study used the analysis of methylation levels by real-time PCR method, MS-HRM, in addition in the present study the whole sample was used.

Also, in regard to the associations found in this study among *ADRB3* DNA hypermethylation, an increased likelihood of being overweight or obese, and a higher waist–hip ratio for men, It has been suggest that the expression of the *ADRB3* gene, which codes for the β-adrenergic receptor, was significantly reduced in fat tissue of obese individuals [14]. In regard to the waist–hip ratio, Guay et al. [2] also found that *ADRB3* hypermethylation was associated with a higher waist–hip ratio in men with a diagnosis of hypercholesterolemia.

Further studies must be conducted because the waist–hip ratio better defines abdominal obesity which. Men have more visceral fat, and women have more subcutaneous fat due to the effects of sexual hormones, which play an important role in the regulation of body fat distribution [39], although the molecular mechanisms involved remain obscure. More work is needed to be investing the association between genetics and sexual hormones [40].

Another relevant finding of this study was the correlation of hypermethylation with regular trans-fatty acid (TFA) intake, even with a median intake below the recommended allowance [41]. There are no studies available for comparative purposes, but it is thought that the intake of this type of fatty acids may be associated with the development and worsening of cardiovascular disease [42].

There was no association found between *ADRB3* methylation and total energy or nutrient intake, but the food ingested by the majority of the population studied was within the recommended range based on the calculation of the prevalence of inadequate consumed dietary components, noting that calorie requirements were calculated individually for the whole population based on the variables gender, age, physical activity, weight, and height [28]. For other nutrients, the intake was lower or within the recommended range for more than 60% of the population. A high prevalence of inadequate folate intake was observed, affecting 88% of the studied population, and no association between *ADRB3* hypermethylation and folate intake was observed.

However, regarding the individuals who perform physical activity, less than 50% classified as active based on the American College of Sports Medicine [43], and the majority had a healthy lifestyle considering the other lifestyle variables.

The association of *ADRB3* hypermethylation with the blood lipid profile was also investigated, and a significant association of hypermethylated individuals presenting higher total cholesterol, LDL-C, and triglyceride levels was observed, but no association with HDL-C was seen. These findings agree with studies conducted by Guay et al. [2], who justify this association with the fact that the *ADRB3* gene is a potential candidate for dyslipidemia.

No significant association was found with homocysteine, and homocysteine levels above the reference values were not observed in this study, which may explain the absence of an association.

Folic acid intake was within the recommended values and showed an association with individuals with hypermethylated *ADRB3*, as found by Claycombe et al. [44] folic acid is a well-known methyl donor and thus has a relevant impact on the methylation profile [45].

Oxidative stress was also associated with *ADRB3* hypermethylation, but not to a level of significance. However, the levels of the oxidative stress indicators used in the present study, TAC and MDA, were within the reference range. In the literature, there are only reports of the induction of DNA methylation changes by the inflammatory process [46, 47].

With regard to the function of *ADRB3* in energy intake, lipid profile, and folic acid levels, the study of epigenetic variation in this gene may contribute to a better understanding of the molecular basis of obesity and cardiovascular diseases.

## Conclusions

In this first analysis of DNA methylation of the *ADRB3* gene in a population-based study, the most relevant results obtained were the high prevalence and association between *ADRB3* DNA hypermethylation with overweight and obesity in both genders, a higher waist–hip ratio for men, higher trans-fat intake, an altered lipid profile, and decreased folic acid intake.

Therefore, considering that DNA methylation may have an impact in adulthood because of its persistence, in particular in regard to the development of non-communicable diseases, data from this study may help in the use of DNA methylation as an effective biomarker. Moreover, the study of epigenetic changes may contribute to a better understanding of the frequently observed associations between lifestyle factors, genetics, and metabolic disorders. However, additional studies must be conducted to determine if the findings of this study are causal or correlative.

## Abbreviations

MDA: malondialdehyde
TAC: total antioxidant capacity
SAM: *S*-adenosylmethionine
MSM: Multiple Source Method
TFA: trans-fatty acid
HPLC: high-performance liquid chromatography
MSP: methylation-specific PCR
TBA: thiobarbituric acid

## References

[1] Schubeler D (2015). Function and information content of DNA methylation. Nature.

[2] Minarovits J, Banati F, Szenthe K, Niller HH (2016). Epigenetic regulation. Adv Exp Med Biol.

[3] Guay SP, Voisin G, Brisson D, Munger J, Lamarche B, Gaudet D (2012). Epigenome-wide analysis in familial hypercholesterolemia identified new loci associated with high-density lipoprotein cholesterol concentration. Epigenomics..

[4] Guay SP, Brisson D, Lamarche B, Biron S, Lescelleur O, Biertho L (2014). ADRB3 gene promoter DNA methylation in blood and visceral adipose tissue is associated with metabolic disturbances in men. Epigenomics.

[5] Martinez JA, Fermín IM, Claycombe JK, Schalinske KL (2014). Epigenetics in adipose tissue, obesity, weight loss, and diabetes. Adv Nutr.

[6] Holliday R (2006). Epigenetics: a historical overview. Epigenetics.

[7] Zheng J, Xiao X, Zhang Q, Yu M (2014). DNA methylation: the pivotal interaction between early-life nutrition and glucose metabolism in later life. Br J Nutr.

[8] Bishop KS, Ferguson LR (2015). The interaction between epigenetics, nutrition and the development of cancer. Nutrients.

[9] Gilbert ER, Liu D (2012). Epigenetics: the missing link to understanding beta-cell dysfunction in the pathogenesis of type 2 diabetes. Epigenetics.

[10] Milagro FI, Campión J, García-Díaz DF, Goyenechea E, Paternain L, Martínez JA (2009). High fat diet-induced obesity modifies the methylation pattern of leptin promoter in rats. J Physiol Biochem.

[11] Dias MM, Souza FR, Takada L, Feitosa FL, Costa RB, Diaz ID, Cardoso DF (2015). Study of lipid metabolism-related genes as candidate genes of sexual precocity in Nellore Cattle. Genet Mol Res.

[12] Lonnqvist F, Thome A, Nilsell K, Hoffstedt J, Arner P (1995). A pathogenic role of visceral fat beta 3-adrenoceptors in obesity. J Clin Investig.

[13] Yu XY, Lin SG, Wang XM, Liu Y, Zhang B, Lin QX (2007). Evidence for coexistence of three beta-adrenoceptor subtypes in human peripheral lymphocytes. Clin Pharmacol Ther.

[14] Kurylowicz A, Jonas M, Lisik W, Jonas M, Wicik ZA, Wierzbicki Z (2015). Obesity is associated with a decrease in expression but not with the hypermethylation of thermogenesis-related genes in adipose tissues. J Transl Med.

[15] Lillycrop KA, Hoile SP, Grenfell L, Burdge GC (2014). DNA methylation, ageing and the influence of early life nutrition. Proc Nutr Soc..

[16] Do Amaral CL, Milagro FI, Curi R, Martínez JA (2014). DNA methylation pattern in overweight women under an energy-restricted diet supplemented with fish oil. Biomed Res Int..

[17] IBGE.Instituto Brasileiro de Geografia E Estatística (2010). Contagem da população 2010.

[18] Kac G, Sichieri R, Gigante DP (2007). Epidemiologia *Nutricional*.

[19] Cochran WG (1977). Sampling techniques.

[20] World Health Organization (2000). Obesity: preventing and managing the global epidemic. Report of a WHO consultation. World Health Organ Tech Rep Ser..

[21] Grundy SM, Cleeman JI, Daniele SR (2005). Diagnosis and management of the metabolic syndrome. Circulation.

[22] Lima LP, Sampaio HAC (2007). Caracterização socioeconômica, antropométrica e alimentar de obesos graves. Ciência Saúde Coletiva.

[23] Lima FEL, Latorre MRDO, Costa MJC, Fisberg RM (2008). Diet and cancer in Northeast Brazil: evaluation of eating habits and food group consumption in relation to breast cancer. Cadernos de Saúde Pública.

[24] Asciutti LSR, Rivera MAA, Costa MJC, Imperiano E, Arruda MS, Bandeira MG (2005). Manual de porções média em tamanho real baseado no programa Dietsys para estudo de base populacional.

[25] Pereira DDEC, Lima RP, De Lima RT, Gonçalves MDAC, De Morais LC, Franceschini SDOC (2013). Association between obesity and calcium: phosphorus ratio in the habitual diets of adults in a city of Northeastern Brazil: an epidemiological study. Nutrition.

[26] Costa MJC, Guilland JC, Moreau D, Boggio V (2008). Vitamin status of health subjects in burgundy (France). Ann Nutr Metab.

[27] MSM—The Multiple Source Method. Department of Epidemiology of the German Institute of Human Nutrition Potsdam–Rehbrucke. https://msm.dife.de/tps/msm/. Accessed 17 Nov 2014.

[28] Otten JJ, Hellwig JP, Meyers LD (2006). Dietary reference intakes: the essential guide to nutrient requirements.

[29] Friedewald WT, Levy RI, Fredrickson DS (1972). Estimation of the concentration of low-density lipoprotein cholesterol in plasma, without use of the preparative ultracentrifuge. Clin Chem.

[30] Ohkawa H, Ohishi N, Yagi K (1979). Assay for lipid peroxides in animal tissues by thiobarbituric acid reaction. Anal Biochem.

[31] Pfeiffer CM, Huff DL, Gunter EW (1999). Rapid and accurate HPLC assay for plasma total homocysteine and cysteine in a clinical laboratory setting. Clin Chem.

[32] Siegel S (1977). Estatística não paramétrica para as ciências do comportamento.

[33] StataCorp (2013). Stata Statistical Software: Release 13.

[34] Thompson RF, Atzmon G, Gheorghe C (2010). Tissue-specific dysregulation of DNA methylation in aging. Aging Cell.

[35] de Oliveira SR, da Silva IC, Mariz BA, Pereira AM, de Oliveira NF (2015). DNA methylation analysis of cancer-related genes in oral epithelial cells of healthy smokers. Arch Oral Biol.

[36] Bezerra SFO, Costa LA, Freitas PAN, Oliveira NFP (2014). Age-related changes in DNA methylation status of hTERT gene promoter of oral epithelial cells. Braz Arch Biol Technol.

[37] El-Maarri O, Becker T, Junen J, Manzoor SS, Diaz-Lacava A, Schwaab R (2007). Gender specific differences in levels of DNA methylation at selected loci from human total blood: a tendency toward higher methylation levels in males. Hum Genet.

[38] Oliveira Y, Lima RPA, Luna RCP (2018). Decrease of the DNA methylation levels of the ADRB3 gene in leukocytes is related with serum folate in eutrophic adults. J Transl Med.

[39] Wells JC (2007). Sexual dimorphism of body composition. Best Pract Res Clin Endocrinol Metab.

[40] Wen W, Kato N, Hwang JY, Xingyi G, Yasuharu T, Huaixing LI (2016). Genome-wide association studies in East Asians identify new loci for waist–hip ratio and waist circumference. Sci Rep..

[41] Cozzolino SMF. Biodisponibilidade de nutrientes. Ed. Manole, 2ª Edição. São Paulo; 2007.

[42] FDA—Food and Drug Administration. FDA Takes step to remove artificial trans fats from processed foods. 2015. https://www.fda.gov/Food/LabelingNutrition/ucm073621.htm.

[43] American College of Sports Medicine (2011). Disponible http://www.acsm.org/docs/fit-society-page/2011summerfspn_behaviorchange.pdf. Accessed 03 Jan 2016.

[44] Claycombe KJ, Brissete CA, Ghrib O (2015). Epigenetics of inflammation, maternal infection, and nutrition. J Nutr..

[45] Yara S, Lavoie JC, Levy E (2015). Oxidative stress and DNA methylation regulation in the metabolic syndrome. Epigenomics.

[46] Huang SK, Scruggs AM, Donaghy J, McEachin RC, Fisher AS, Richardson BC (2012). Prostaglandin E (2) increases fibroblast gene-specific and global DNA methylation via increased DNA methyltransferase expression. FASEB J.

[47] Einstein F, Thompson RF, Bhagat TD (2010). Cytosine methylation dysregulation in neonates following intrauterine growth restriction. PLoS ONE..

[48] WHO-World (1999). Definition, diagnosis and classification of diabetes mellitus and its complications. Report of a WHO consultation.

[49] American Heart Association (AHA). Circulation. 2010;121:948–954. 10.1161/CIRCULATIONAHA.109.192666 20177011

[50] USDA-Department of Agriculture. Report of the Dietary Guidelines Advisory Committee on the Dietary Guidelines for Americans: to the Secretary of Agriculture and the Secretary of Health and Human Services. Washington, DC: USDA and US DHHS, 2010.

